# Panchromatic Fluorescence Emission from Thienosquaraines Dyes: White Light Electrofluorochromic Devices

**DOI:** 10.3390/molecules26226818

**Published:** 2021-11-11

**Authors:** Giuseppina Anna Corrente, Francesco Parisi, Vito Maltese, Sante Cospito, Daniela Imbardelli, Massimo La Deda, Amerigo Beneduci

**Affiliations:** 1 Laboratory of Physical Chemistry, Materials and Processes for Industry, Environment and Cultural Heritage, Department of Chemistry and Chemical Technologies, University of Calabria, Via P. Bucci, Cubo 15D, 87036 Arcavacata di Rende, Italy; giuseppina.corrente@unical.it (G.A.C.); vito.maltese@unical.it (V.M.); sante.cospito@unical.it (S.C.); daniela.imbardelli@unical.it (D.I.); 2 Laboratory of Inorganic Molecular Materials, Department of Chemistry and Chemical Technologies, Institute of Nanotechnology CNR-Nanotec, University of Calabria, Via P. Bucci, Cubo 14C, 87036 Arcavacata di Rende, Italy; francesco.parisi@unical.it (F.P.); massimo.ladeda@unical.it (M.L.D.)

**Keywords:** squaraines, photoluminescence, electrofluorochromism, electrofluorochromic device, electrochemical properties

## Abstract

Electrofluorochromic devices (EFCDs) that allow the modulation of the light emitted by electroactive fluorophores are very attractive in the research field of optoelectronics. Here, the electrofluorochromic behaviour of a series of squaraine dyes was studied for the first time. In solutions, all compounds are photoluminescent with maxima located in the range 665–690 nm, characterized by quantum yields ranging from 30% to 4.1%. Squaraines were incorporated in a polymer gel used as an active layer in all-in-one gel switchable EFCDs. An aggregation induced quenching occurs in the gel phase, causing a significant decrease in the emission quantum yield in the device. However, the squaraines containing the thieno groups (thienosquaraines, TSQs) show a panchromatic emission and their electrofluorochromism allows the tuning of the fluorescence intensity from 500 nm to the near infrared. Indeed, the application of a potential difference to the device induces a reversible quenching of their emission that is significantly higher and occurs at shorter switching times for TSQs-based devices compared to the reference squaraine dye (DIBSQ). Interestingly, the TSQs fluorescence spectral profile becomes more structured under voltage, and this could be explained by the shift of the aggregates/monomer equilibrium toward the monomeric species, due to electrochemical oxidation, which causes the disassembling of aggregates. This effect may be used to modulate the colour of the fluorescence light emitted by a device and paves the way for conceiving new electrofluorochromic materials based on this mechanism.

## 1. Introduction

Stimulus-responsive materials have attracted significant attention due to their potential applications in the fields of data storage, sensors, biomedicine, and information displays [1,2,3,4,5,6]. It is well known that the main external stimuli include temperature, light, mechanical force, solvent, and electric or magnetic fields [7,8,9,10,11,12,13,14,15,16]. Among these, the electrical stimulus is particularly important in optical electronic devices [17,18,19,20,21]. Electrofluorochromism (EFC) refers to the reversible tuning of the fluorescent properties of a material, such as emission intensity, wavelength, and lifetime, under electrical stimulus. The development of novel EFC materials is very important since these can be applied to various multifunctional and intelligent devices. The EFC materials, mainly belong to two classes: molecular dyads (a fluorophore is linked to a redox unit which acts as a quencher through photoinduced electron/energy transfer processes between the excited state of the fluorophore and the redox unit) and redox active fluorophores (a change of the fluorescence emission is caused by a direct oxidation or reduction of the fluorophore) [22,23,24].

In this last case, the reversibility of the electrofluorochromic strongly depends on the stability of the radical ion formed during the redox process. 

**Figure 1:**
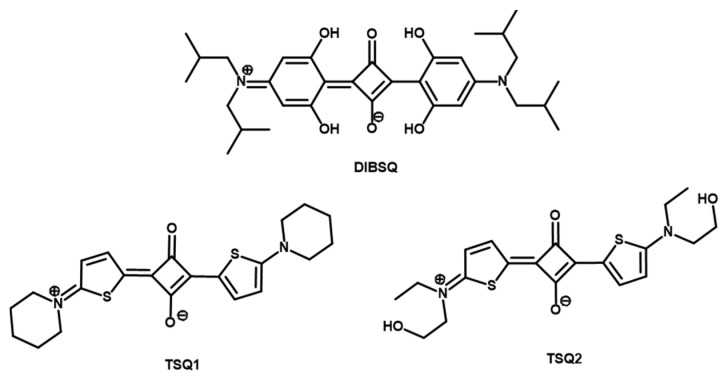
Molecular structures of investigated squaraine and thienosquaraines.

1,3-donor disubstituted squaraines (SQs) are an emergent class of donor–acceptor–donor (D–A–D) chromophores with a unique resonance-stabilized zwitterionic structure, in which electrons are highly delocalized over the conjugated bridge [25,26,27]. For this reason, SQs exhibit several chemical-physical properties such as strong absorption in the visible and near infrared (NIR) regions and excellent photochemical and photophysical stability [26,27,28,29]. All this makes these organic dyes very promising multifunctional materials for a wide range of applications, including photovoltaics [26,28,30,31], biological imaging [27,28,29,32,33,34], chemo/biosensors [27,28,29], photodynamic and photothermal therapy [28,29,35,36,37], and optical communication technology [38]. Moreover, SQs are molecular switches that can be reversibly shifted between two or more stable redox states in response to an electrochemical potential, therefore their optical properties can be easily modulated [38,39,40,41]. So far, few scientific works [38,39,40,41] have investigated the ability of these dyes to change their absorption spectrum, a phenomenon well known as electrochromism [42,43,44]. Instead, no one has yet explored their electrofluorochromic behaviour, which consists of the modulation of photoluminescence intensity [22,24,45]. In this work, we present for the first time an electrofluorochromic study based on squaraines and thienosquaraines (Figure 1). More specifically, we assembled all-in-one gel devices with the commercial 2,4-bis[4-(N,N-diisobutylamino)-2,6-dihydroxyphenyl] squaraine (DIBSQ) coupled with ferrocene and the synthetized thienosquaraines (TSQ1 and TSQ2, Figure 1) with the ethyl viologen, that were characterized by extensive electrofluorochromic study in order to disclose their potential applicability. Particularly, we studied the devices at different operating voltage, highlighting an interesting tuning of the electro-optical properties of device components. Compared to “classic” squaraines, thienosquaraines exhibit panchromatic fluorescence emission, by which devices that emit white light can be used to modulate its intensity in almost the whole visible spectral range.

## 2. Results and Discussion

### 2.1. Electrochemical Properties

The electrochemical properties in dichloromethane solution of the squaraines investigated here are reported in Table 1 [40]. The thienosquaraines can be easily oxidized to form the radical cation species, which is stabilized by charge delocalization over the core of the molecule and around the nitrogen atoms. The higher oxidation potential of DIBSQ is due to a relatively less stabilization effect of the radical cation by charge delocalization. The squaraines can be also reduced to the radical anion species, where the negative charge in TSQs is more concentrated on the cyclobutadienyl ring, while, in DIBSQ it is better stabilized by delocalization over the core and the phenyl rings [40]. This is reflected in the substantial different spectroelectrochemical behaviour of these squaraines. Indeed, DIBSQ shows electrochromism in reduction, whereas the thienosquaraines show electrochromism in oxidation [40].

### 2.2. Photophysical Properties in Solution

The photophysical characterization of TSQ1 and TSQ2 has been performed in chloroform solution, and the obtained results have been compared with the photophysics of DIBSQ. Figure S1 in SI reports the absorption spectra of the three compounds, which exhibit cyanine-like sharp absorption bands in the visible-NIR part of the spectrum between 650–665 nm that can be assigned to the π-π* transition, whereas the shoulder located at higher energies can be interpreted as a vibronic replica [40,46]. All compounds are luminescent, with a small Stokes shift of the emission bands (Figure S2 in ESI), where maxima are located in the 665–690 nm range, while some shoulders are detected at longer wavelengths, showing a correspondence with the absorption spectra, of which they are an excellent mirror image. Despite the remarkable similarity of the spectra, the emission quantum yield of the thienosquaraines is considerably lower than that of DIBSQ. In fact, while the yield of the latter has a value of 30%, in the case of TSQ1 and TSQ2 the value is, respectively, 4.1 and 6.0%. This can be attributed to an increase in the non-radiative rate constant of thienosquaraines.

### 2.3. Photophysical Properties in Device

**Figure 2:**
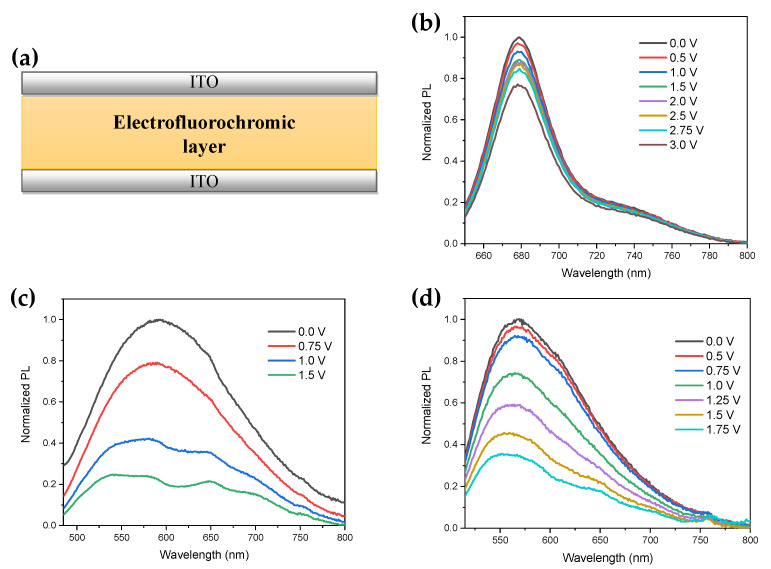
Electrofluorochromism of ITO/EFC/ITO devices, schematized in panel (**a**), containing the (**b**) DIBSQ/FC; (**c**) TSQ1/EV (**d**) TSQ2/EV systems. Film thickness = 15 μm.

The fluorescence properties of the squaraines were also studied in the device, which was fabricated by incorporating the electroactive components in a thermoplastic polymer gel in order to form an all-in-one solid gel electrofluorochromic layer. The electroactive gel was, indeed, laminated at 80 °C between two Indium-tin-oxide (ITO) coated glass substrates at a thickness of 15 μm [18,20,21]. The architecture of the device is depicted in Figure 2a.

#### 2.3.1. Photophysical Properties in the off State

**Figure 3:**
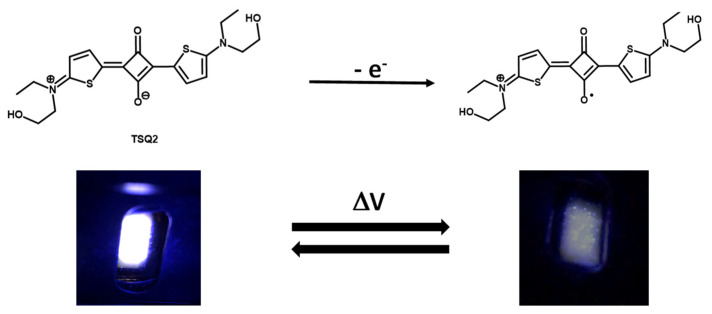
Electrochemical oxidation of the thienosquaraine TSQ2 in the device: the white fluorescence of the zwitterionic species is quenched by the application of a potential difference that generate the low-emissive radical cation species.

The fluorescence spectra of the devices as a function of the potential difference applied are displayed in Figure 2b–d. In the off state, i.e., at zero voltage, the fluorescence spectrum of the DIBSQ shows a relatively intense band centred at 678 nm and another very low intensity emission at longer wavelengths, according to its fluorescence spectrum in solution (Figure 2b). Thus, the emission spectrum of DIBSQ in the gel phase is only slightly red-shifted (12 nm) and slightly broadened (FWHM 3.5 nm larger) with respect to that in solution, thus remaining rather narrow (Figure S3 and Tables S1 and S2). These features could be explained by the formation of J-aggregates in the gel phase between the DIBSQ molecules [47], though the quantum emission yield shows a considerable decrease, down to 0.04%. In contrast, the TSQs show a wide emission band covering almost the whole visible spectrum (panchromatic emission), leading to white light emission (Figure 3, see also the CIE diagram in Figure S4), and the maximum emission wavelength is strongly blue-shifted compared to the spectra in solution. These fluorescence spectral features, which are rather anomalous for squaraine fluorophores, are reasonably due to a strong aggregation of the thienosquaraines in the gel phase. In general, squaraines show a high tendency to aggregation due to their planar structure, favouring π-π stacking and electrostatic interactions, leading to either J- or H-aggregates, both having red-shifted emission [48,49,50]. We have already shown that the thienosquaraines do indeed aggregate in concentrated solution and in the solid state, leading to very broad absorption spectra compared to those in a dilute solution. Moreover, the spectra of the aggregates are characterized by broad and rather intense bands in the 400–500 nm range, and by rather narrow, though less intense, bands in the near infrared range [40]. This evidence suggests the formation of aggregates of the two types (H- and J-) for the thienosquaraines. Therefore, the broad (Vis-NIR) and blue-shifted emission of TSQs (Figure 2b,c) may arise from a plethora of different types of aggregates covering a wide range of emission properties. In addition, aggregation may well explain the photoluminescence quenching observed at increasing solution concentration up to the gel phase (Table S3), due to a rapid intermolecular charge transfer between the thienosquaraines molecules [51], that indeed shows quantum yields in the devices of 0.2 and 0.3% for TSQ1 and TSQ2, respectively.

#### 2.3.2. Electrofluorochromic Characterization

Figure 2b–d also shows that upon the application of a potential difference to the device, a reversible fluorescence quenching occurs for all the systems. It suggests that the chromophore backbone is influenced by electrochemical oxidation (reduction for DIBSQ) (Figure 3), which is similar to what happens upon protonation [52]. This effect may explain the reduced photoluminescence quantum yield compared to the system at zero voltage, i.e., the electrofluorochromic effect. Indeed, the fluorescence quenching is caused by the bleaching of the typical absorption band of these compounds arising from an internal charge transfer transition between the olate and the nitrogen atoms, as previously observed [40].

The relatively intense white light emitted by the TSQs devices in the off state is therefore quenched as the low-emissive radical cation species is generated (Figure 3). This mechanism is reversible upon potential inversion (Video S1).

Interestingly, the fluorescence spectrum of the thienosquaraines becomes more structured at increasing voltages (Figure 2c,d). This could be rationalized by assuming that the elecrochemical oxidation affects the self-assembling of the aggregates, the equilibria among them and with the monomeric form, ultimately leading to their disassembling into the monomer. In order to obtain insights into this hypothesis, we performed a deconvolution of the spectra as a function of the applied voltage, and calculated the contribution of each species (aggregates, monomer) to the overall fluorescence. The deconvoluted spectra are reported in Figures S5 and S6, while Table 2 collects the contribution of each band to the cumulative spectrum acquired at a specific potential difference. For the TSQ1 system we were able to fit the overall spectrum at each potential with three bands, one occurring close to the peak maximum of the monomer in solution (670 nm, Figure S2) and the other two centred at about 600 nm and 540 nm (Figure S5). There is an almost monotonic increase in the relative contribution of the monomer band to the overall spectrum with increasing potential difference, at the expense of the other two bands, which can be assigned to aggregates of different supramolecular structures (indicated as Aggregates I and II in Table 2 for simplicity). This suggests that TSQ1 oxidation causes a significant destructuration of the aggregates, which is voltage dependent since more and more TSQ1 molecules are attracted to the anode and subtracted from the bulk. 

The spectrum of TSQ2 can be well deconvoluted by two bands centred at about 605 nm and 557 nm, indicating an almost negligible contribution of the monomeric species to the overall fluorescence. A possible explanation for this observation may be provided by the presence of the OH groups in the TSQ2 molecule, that could better interact with the polymer matrix (PVF), which may favour the aggregation on its backbone. In contrast, as the potential difference applied to the device is increased, a new band peaked at 660 nm, must be introduced to fit the emission spectrum (Figure S6), suggesting that also in this case, the oxidation of the thienosquaraine partially disrupts the aggregates in the bulk of the cell, in a similar manner as described above for TSQ1.

Table 3 shows the significant voltage-dependent changes in the relative contribution of each band to the overall light emitted by the device, highlighting the fact that there is a continuous modulation of the spectral profile, i.e., of the colour of the light emitted, by virtue of the shift of the aggregates/monomer equilibria induced by the electrochemical oxidation of the squaraines. Thus, this electrofluorochromic mechanism, reported for the first time here, may be advantageously used to modulate the colour of the fluorescence light emitted, and paves the way for conceiving new electrofluorochromic materials based on this mechanism.

The electrofluorochromic effect is significantly higher for TSQs-based devices. Indeed, the observed contrast ratio follows the order TSQ1 (4.8) > TSQ2 (2.9) > DIBSQ (1.3). 

Actually, the electrofluorochromic switching times are relatively high compared to those observed in similar devices, based on charged electroactive species where the gel polymer phase was the same [20,53]. The kinetics of the quenching and emission processes are clearly influenced by the nature of the matrix, incorporating the electroactive species, as well as by the nature of the last ones. In the case of squaraines, it can be argued that the kinetics of the electrofluorochromic effect is largely influenced by the kinetics of the assembling/disassembling aggregates, which, as already highlighted, plays a critical role in the reversible quenching mechanism. Another effect contributing to slowing down the electrofluorochromic response may be due to the zwitterionic nature of these compounds, which probably affects the migration of SQs towards the electrodes in the gel phase. It is interesting to note that the switching times for the quenching process, relative to the TSQs, are generally shorter than those observed for DIBSQ (at the same contrast), which could be explained by the speeding up effect of the EV dictation, which is rapidly reduced at the cathode, leading to an initial net excess of negative charge density on this side of the device. This helps to push the zwitterionic species toward the anode under the action of the potential difference. On the other hand, the switching times for the reverse processes are comparable among the different systems without taking into account the contrast value. 

## 3. Materials and Methods

As previously reported, 2,4-bis[4-(N,N-diisobutylamino)-2,6-dihydroxyphenyl] squaraine (DIBSQ), [54] and 1,3-bis(5-piperidin-1-yl-thiophen-2-yl)squarene [55] and 1,3-bis{5-[ethyl(2-hydroxyethyl)amino]thiophen-2-yl}squarene were synthesized freshly before use [40]. Poly(vinyl formal) (PVF), Ferrocene (Fc) and Ethyl Viologen (EV) were purchased from Sigma Aldrich and were not further purified before use. N-methyl-2-pyrrolidinone, NMP, Pancreac) was used as solvent. 

According to a previous report where it was shown that the DIBSQ works as cathode and the thienosquaraines as anodes in electrochromic devices [40], DIBSQ was coupled to ferrocene and the thienosquaraines to ethylviologen. The DIBSQ/Fc EFC polymer gels were prepared with 2% (*w*/*w*) of DISBQ, 1% (*w*/*w*) of ferrocene, 40% (*w*/*w*) of PVF, and 57% (*w*/*w*) of NMP. The TSQs/EV EFC polymer gels were prepared with 2% (*w*/*w*) of the anodic component, 1% (*w*/*w*) of EV, 40% (*w*/*w*) of PVF and 57% (*w*/*w*) of NMP. The two electroactive species were first dissolved in NMP at room temperature and then mixed with the polymer under continuous stirring at 130 °C for 1 h. 

ITO/EFC gel/ITO devices were assembled by drop casting of the hot EC mixture onto an ITO-coated glass support (Visiontek Systems Ltd. with a sheet resistance of 25 Ω sq^−1^ and a thickness of 1 mm) with a second ITO electrode used to create a sandwich with an active area of about 1.5 × 1.5 cm^2^. The cell gap was controlled by inserting cylindrical spacers with a mean base diameter of 15 × 10^−6^ m [53,56].

The photophysical investigations in solution were performed with spectrofluorimetric grade solvents. The absorption spectra were acquired with Perkin Elmer Lambda 900 spectrophotometer. Steady-state emission spectra were recorded on a HORIBA Jobin-Yvon Fluorolog-3 FL3-211 spectrometer equipped with a 450 W xenon arc lamp, double-grating excitation, and single-grating emission monochromators (2.1 nm/mm dispersion; 1200 grooves/mm), and a Hamamatsu R928 photomultiplier tube. Emission and excitation spectra were corrected for source intensity (lamp and grating) and emission spectral response (detector and grating) by standard correction curves.

The emission quantum yields of the samples were obtained by means of a Labsphere optical Spectralon^®^ integrating sphere (diameter 102 mm), which provides a reflectance > 99% over 400–1500 nm range (>95% within 250–2500 nm). The sphere accessories are made from Teflon (rod and sample holders) or Spectralon (baffle). The sphere is mounted in the optical path of the spectrofluorimeter using, as the excitation source, a 450 W Xenon lamp coupled with a double-grating monochromator for selecting wavelengths. Cylindrical tubes containing the solution samples are placed into the sphere, while the ITO sandwich, containing the sample, is placed into the sphere on a customized temperature-controlled hot stage realized in Teflon by CaLCTec s.r.l. (Rende, Italy), with an uncertainty on the temperature of 1°C. The emission quantum yield was determined by a procedure previously described [54].

## 4. Conclusions

Squaraines dyes are well known for their industrial applications in diverse fields, including photovoltaics, biological imaging, chemo/biosensors, photodynamic and photothermal therapy, optical communication technology. Here, the electrofluorochromic response of different 1,3-donor disubstituted squaraine dyes was reported for the first time, in order to explore the possible application of this class of multifunctional materials in light switch applications. We have shown that, depending on the type of donor substituents on the squarylium core, they can show electrofluorochromism by electrochemical reduction (DIBSQ) and oxidation (TSQs). In both cases, the application of a dc voltage to the device causes a quenching of the photoluminescence of the dyes, which can be restored upon voltage inversion. The PL quenching is due to the bleaching of the typical absorption band of these compounds, arising from an internal charge transfer transition between the olate to the nitrogen atoms, as previously observed. 

Moreover, the thienosquaraines studied show high aggregation in the polymer gel phase, leading to an almost panchromatic emission (white light), which is tuned in intensity and partially in colour, by the application of the voltage. Indeed, the PL spectrum of the quenched species is more structured, revealing a change in the relative contribution of the different aggregates to the overall spectrum. This effect may be used to modulate the colour of the fluorescence light emitted by a device and pave the way for conceiving new electrofluorochromic materials based on this mechanism.

## Figures and Tables

**Table 1:** Electrochemical properties of squaraines dyes in solution and in device.

Compound	^a^ E_g_	^a^ E_ox_	^a^ E_red_	^b^ ΔE
	(eV)	(V)		
DIBSQ	1.66	0.46	−1.20	0.50
TSQ1	1.16	−0.12	−1.28	0.65
TSQ2	0.95	−0.05	−1.00	0.60

^a^ Referenced to the Fc/Fc^+^. Measured in a 0.1 M TBAPF_6_/CH_2_Cl_2_ solution with a dye concentration of 10^−3^ M, at 25 °C. The electrochemical potential of Fc/Fc^+^ was set at 4.8 V versus vacuum. ^b^ Switching potential measured in the CV of the EFC device.

**Table 2:** Contribution of each fluorescence band to the overall emission of TSQs in the gel phase, calculated by the spectral deconvolution at different voltages.

		A%_peak1_ (nm)	A%_peak2_ (nm)	A%_peak3_ (nm)	R^2^	Χ^2^
		Aggregates I	Aggregates II	Monomer		
TSQ1	0.0 V	58 (566)	16 (632)	26 (681)	0.9995	4.4 × 10^−5^
	0.75 V	16 (536)	38 (590)	46 (654)	0.9997	2.2 × 10^−5^
	1.0 V	16 (531)	6 (577)	78 (631)	0.9963	6.5 × 10^−5^
	1.5 V	29 (533)	6 (580)	65 (647)	0.9944	3.4 × 10^−5^
TSQ2	0.0 V	29 (557)	71 (605)		0.9973	3.2 × 10^−4^
	0.75 V	28 (557)	72 (604)		0.9972	28 × 10^−4^
	1.5 V	40 (549)	39 (602)	21 (664)	0.9971	7.0 × 10^−5^
	1.75 V	44 (549)	33 (603)	23 (662)	0.9955	54 × 10^−5^

**Table 3:** Electrofluorochromic switching performances of DIBSQ and TSQs based devices ^a^.

	Pulse Sequence V_1__V_2_(V) - T_1__t_2_ (s)	$I_{\mathrm{OFF}}/I_{\mathrm{ON}}\;\left(\sigma \right)$	$\tau_{1}\;\left(\sigma \right)\;(s)$	$\tau_{2}\;\left(\sigma \right)\;(s)$
DIBSQ/Fc	-2_2–60_60	1.52 (0.07)	58 (1)	22 (2)
	2_-2–60_60	1.8 (0.1)	59 (1)	17 (2)
TSQ1/EV	2_-2–60_60	3 (0.2)	47 (2)	20 (2)
TSQ2/EV	2_-2–40_60	1.55 (0.04)	32 (1)	49 (1)

^a^ All the values are the mean and standard deviation (σ) calculated over 50 cycles and three device replicates.

## Data Availability

Not applicable.

## Supplementary Materials

The following are available online, Figure S1. Absorption spectra in chloroform solution, Figure S2. Emission and excitation spectra in chloroform solution: (a) DIBSQ; (b) TSQ1; (c) TSQ2, Figure S3. Normalized fluorescence spectra of DIBSQ in solution and in the polymer gel phase (solid lines) and the deconvolute spectral bands (dotted lines), Figure S4. CIE diagram showing the CIE coordinates for DIBSQ and the two thienosquaraines TSQ1 and TSQ2 at zero voltage, in the devices, Figure S5. Deconvolution of the emission spectra of TSQ1-based device at different voltages: (a) 0.0V, (b) 0.75V, (c) 1.0V and (d) 1.5V, Figure S6. Deconvolution of the emission spectra of TSQ2-based device at different voltages: (a) 0.0V, (b) 0.75V, (c) 1.5V and (d) 1.75V, Table S1. Spectral deconvolution fitting results for the DIBSQ in solution, Table S2. Spectral deconvolution fitting results for the DIBSQ in gel, Table S3. Dependence of the EQY on the dye concentration in CH2Cl2 solution, Video S1.

## Sample Availability

Samples of the compounds are not available from the authors.
