# Supplementary material for: Panchromatic Fluorescence Emission from Thienosquaraines Dyes: White Light Electrofluorochromic Devices
Source: Molecules. 2021 Nov 11;26(22):6818. doi: 10.3390/molecules26226818 (PMC8621610; doi:10.3390/molecules26226818)
Supplement: Supplementary file 1 [file molecules-26-06818-s001.zip › molecules-1433851-supplementary.pdf]

Panchromatic fluorescence emission from thienosquaraines dyes: white light electrofluorochromic devices.

Giuseppina Anna Corrente [1], Francesco Parisi [2], Vito Maltese [1], Sante Cospito [1], Daniela Imbardelli [1], Massimo La Deda [2], Amerigo Beneduci [1,*]

[1] Laboratory of Physical Chemistry, Materials and Processes for Industry, Environment and Cultural Heritage, Department of Chemistry and Chemical Technologies, University of Calabria, Via P. Bucci, Cubo 15D, 87036 Arcavacata di Rende, CS, Italy; giuseppina.corrente@unical.it, vito.maltese@unical.it, sante.cospito@unical.it, daniela.imbadelli@unical.it, amerigo.beneduci@unical.it.
[2] Laboratory of Inorganic Molecular Materials, department of Chemistry and Chemical Technologies, University of Calabria, Via P. Bucci, Cubo 14C, 87036 Arcavacata di Rende, CS, Italy; francesco.parisi@unical.it, massimo.ladeda@unical.it.
* Correspondence: amerigo.beneduci@unical.it.

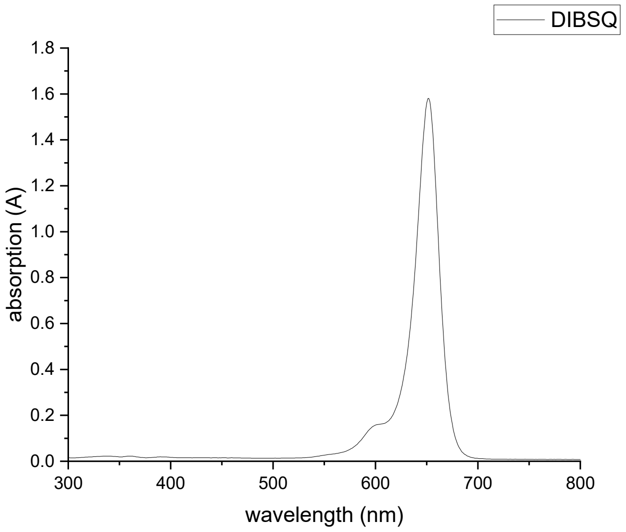

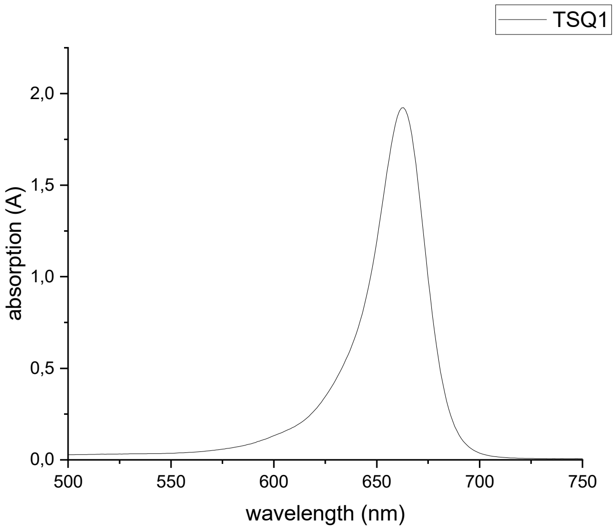

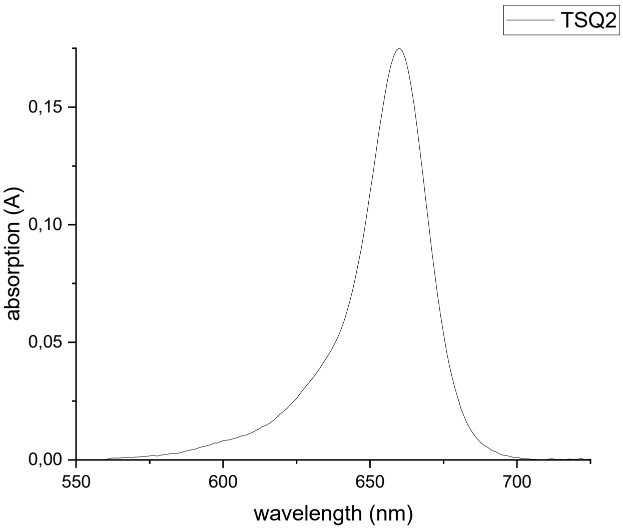

Figure S1. Absorption spectra in chloroform solution.

a)

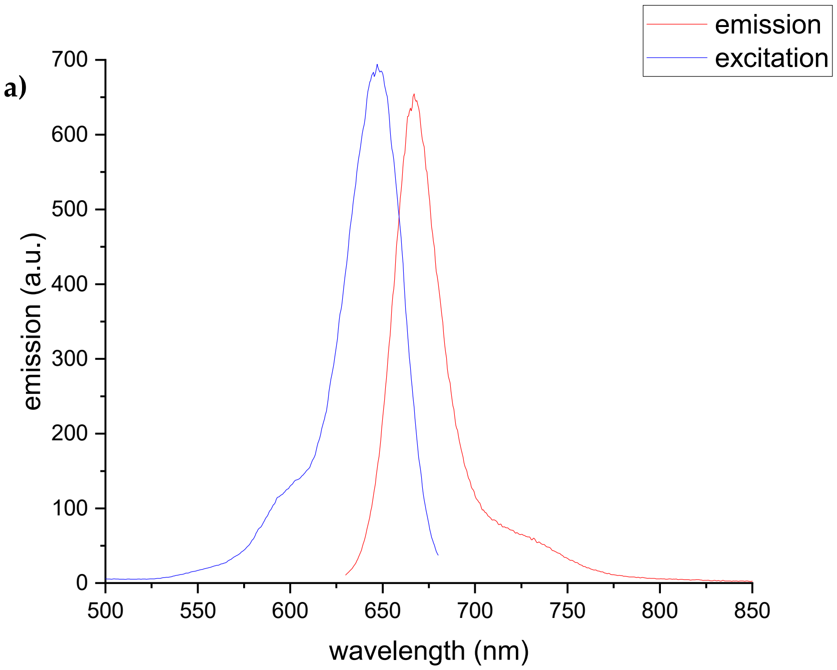

b)

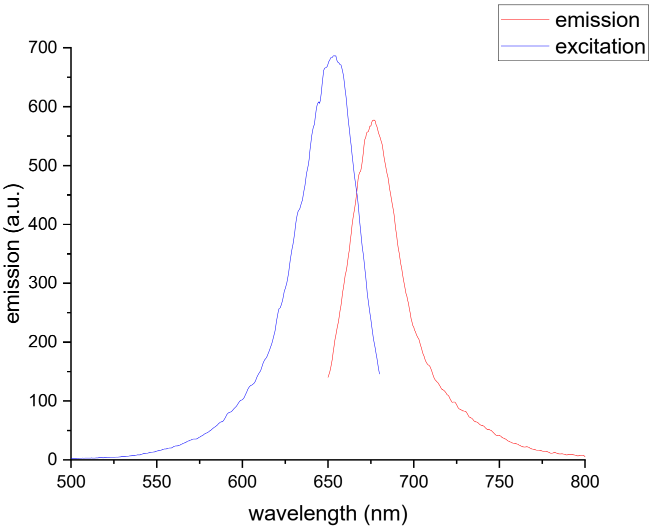

c)

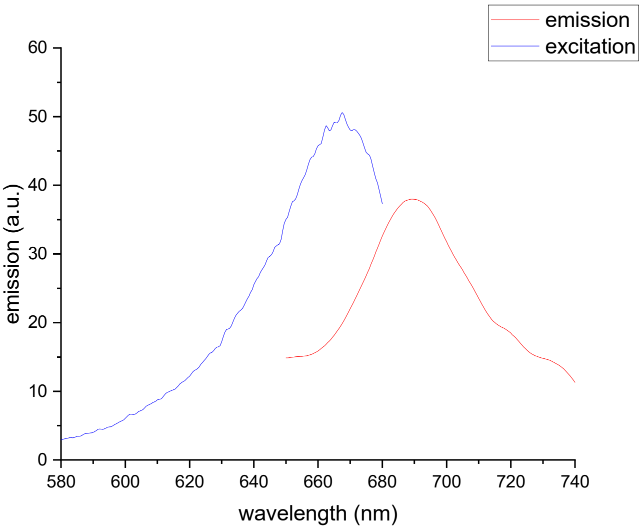

Figure S2. Emission and excitation spectra in chloroform solution: a) DIBSQ; b) TSQ1; c) TSQ2.

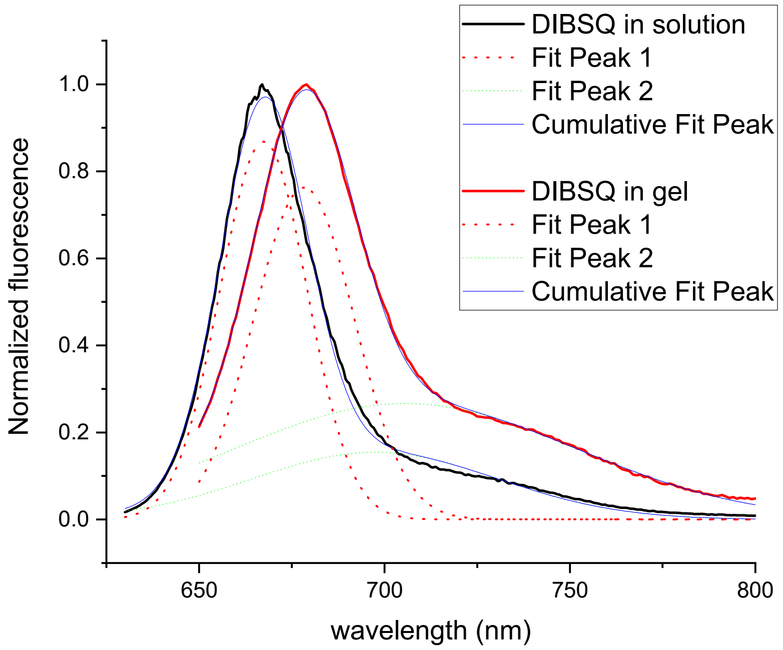

Figure S3. Normalized fluorescence spectra of DIBSQ in solution and in the polymer gel phase (solid lines) and the deconvolute spectral bands (dotted lines).

Table S1. Spectral deconvolution fitting results for the DIBSQ in solution

Model	Gauss	
Equation	y=y0 + (A/(w*sqrt(pi/2)))*exp(-2*((x-xc)/w)^2)	
Plot	Peak1	Peak2
y0	0 ± 0	0 ± 0
xc	667.39607 ± 0.0414	697.42555 ± 0.95667
w	23.57581 ± 0.13117	66.15333 ± 1.06916
A	25.64956 ± 0.25505	12.78607 ± 0.32083
Reduced Chi-Sqr	1.29198E-4	
R-Square (COD)	0.99794	
Adj. R-Square	0.99792	

Table S2. Spectral deconvolution fitting results for the DIBSQ in gel

Model	Gauss	
Equation	y=y0 + (A/(w*sqrt(pi/2)))*exp(-2*((x-xc)/w)^2)	
Plot	Peak1	Peak2
y0	0 ± 0	0 ± 0
xc	678.29111 ± 0.05504	705.62963 ± 0.89136
w	27.11419 ± 0.17044	92.79719 ± 0.97604
A	25.91569 ± 0.30964	30.98553 ± 0.54431
Reduced Chi-Sqr	4.05389E-5	

R-Square (COD)	0.99953	
Adj. R-Square	0.99952	

Table S3. Dependence of the photoluminescence on the dye concentration in CH_2Cl_2 solution

DIBSQ			TSQ1	TSQ2
Conc. (mol/L)	EQY (%)	Conc. (mol/L)	EQY (%)	
2.00×10^{-5}	30.00	1.00×10^{-5}	4.10	6.00
2.00×10^{-4}	24.50	1.00×10^{-4}	2.80	4.10
1.00×10^{-3}	15.00			
3.30×10^{-2} (Gel)[a]	0.04	4.40×10^{-2} (Gel)[a]	0.20	0.30

[a]In NMP solvent

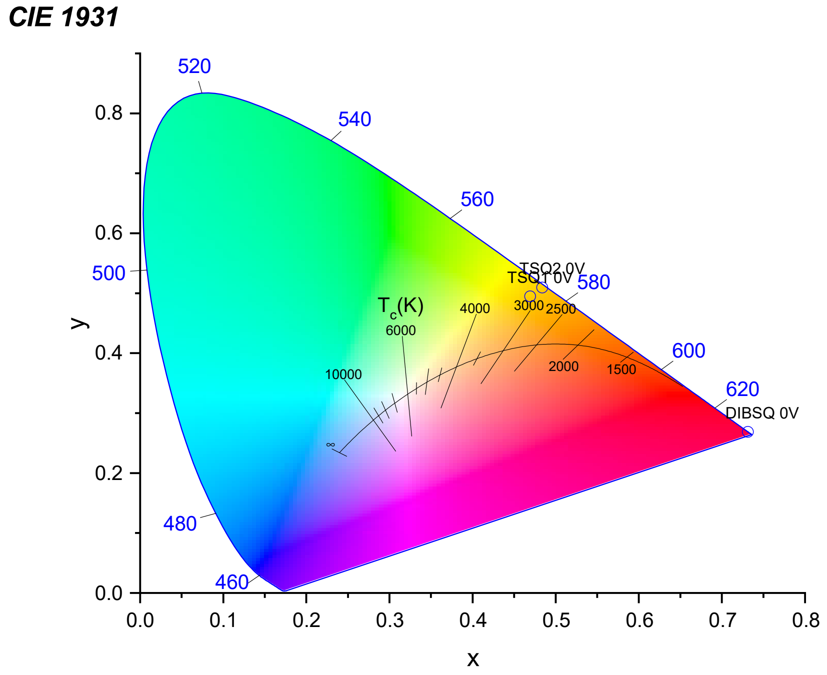

Figure S4. CIE diagram showing the CIE coordinates for DIBSQ and the two thienosquaraines TSQ1 and TSQ2 at zero voltage, in the devices.

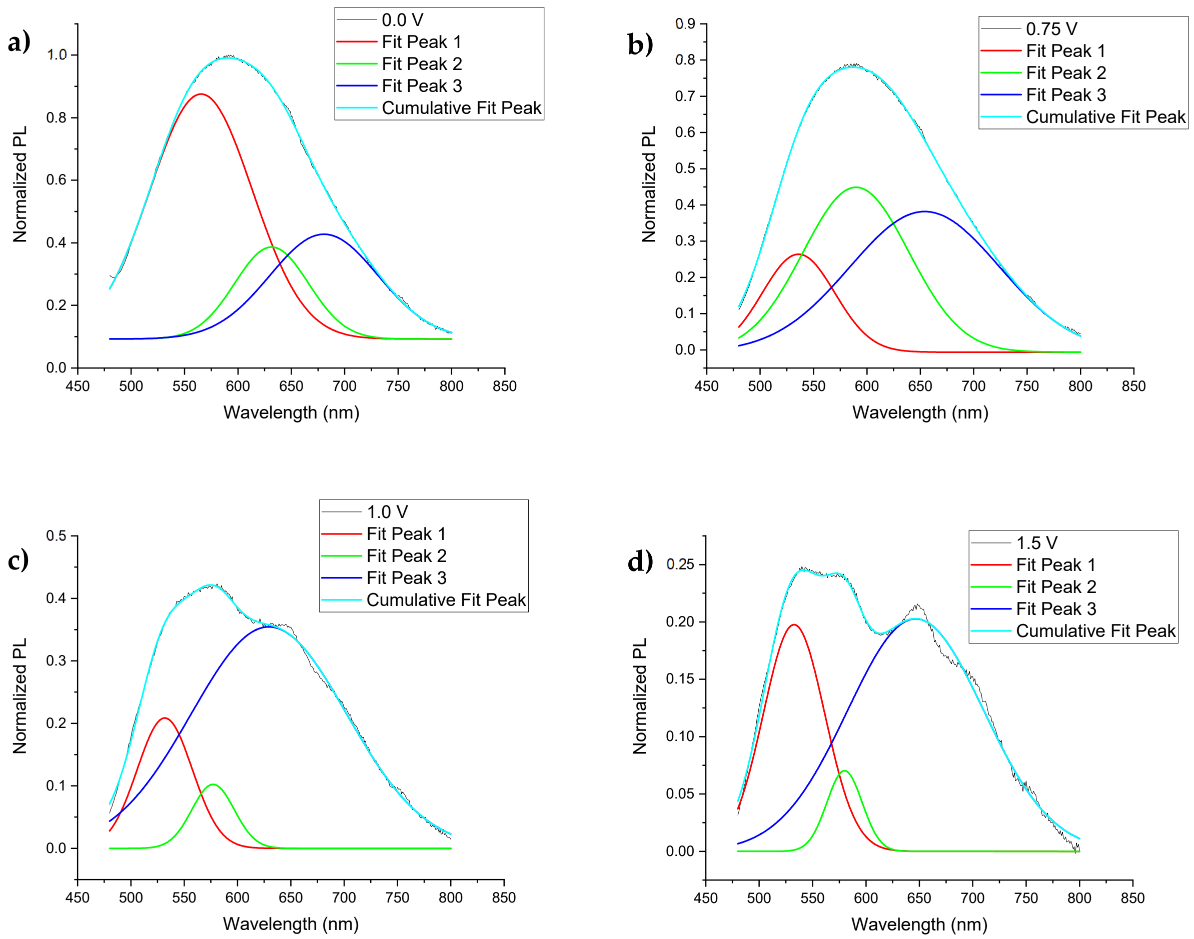

Figure S5. Deconvolution of the emission spectra of TSQ1-based device at different voltages: a) 0.0V, b) 0.75V, c) 1.0V and d) 1.5V.

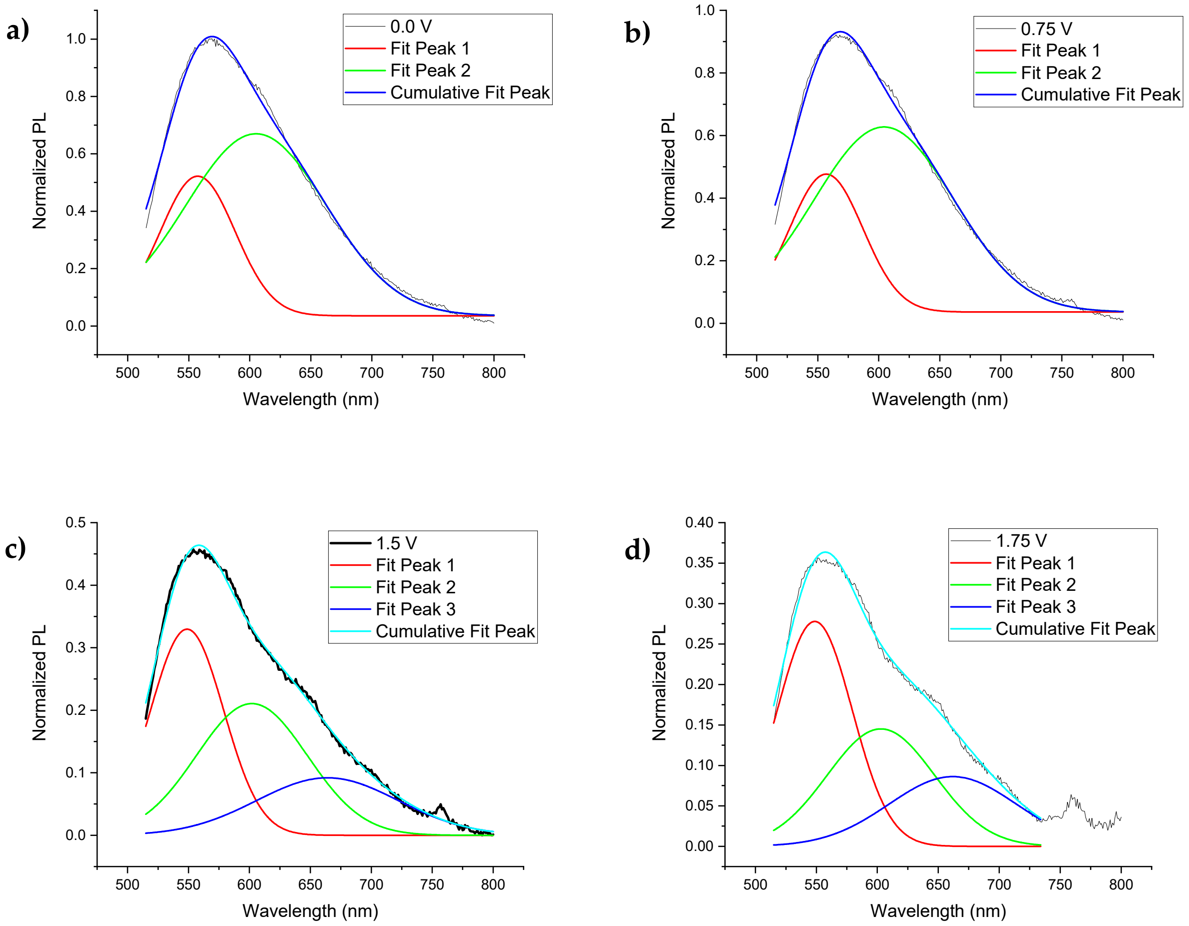

Figure S6. Deconvolution of the emission spectra of TSQ2-based device at different voltages: a) 0.0V, b) 0.75V, c) 1.5V and d) 1.75V.
